# Mutation spectrum of *MLL2 *in a cohort of kabuki syndrome patients

**DOI:** 10.1186/1750-1172-6-38

**Published:** 2011-06-09

**Authors:** Lucia Micale, Bartolomeo Augello, Carmela Fusco, Angelo Selicorni, Maria N Loviglio, Margherita Cirillo Silengo, Alexandre Reymond, Barbara Gumiero, Federica Zucchetti, Ester V D'Addetta, Elga Belligni, Alessia Calcagnì, Maria C Digilio, Bruno Dallapiccola, Francesca Faravelli, Francesca Forzano, Maria Accadia, Aldo Bonfante, Maurizio Clementi, Cecilia Daolio, Sofia Douzgou, Paola Ferrari, Rita Fischetto, Livia Garavelli, Elisabetta Lapi, Teresa Mattina, Daniela Melis, Maria G Patricelli, Manuela Priolo, Paolo Prontera, Alessandra Renieri, Maria A Mencarelli, Gioacchino Scarano, Matteo della Monica, Benedetta Toschi, Licia Turolla, Alessandra Vancini, Adriana Zatterale, Orazio Gabrielli, Leopoldo Zelante, Giuseppe Merla

**Affiliations:** 1 Medical Genetics Unit, IRCCS Casa Sollievo della Sofferenza Hospital, 71013 San Giovanni Rotondo, Italy; 2 Ambulatorio Genetica Clinica Pediatrica, Clinica Pediatrica Università Milano Bicocca, Fondazione MBBM AOS Gerardo Monza, Italy; 3 Dipartimento di Scienze Pediatriche, Università di Torino, Italy; 4 Center for Integrative Genomics, University of Lausanne, Lausanne, Switzerland; 5 Medical Genetics, Bambino Gesù Paediatric Hospital, IRCCS, Rome, Italy; 6 Division of Medical Genetics, Galliera Hospital, Genova, Italy; 7 Laboratory of Medical Genetics, "V.Fazzi" Hospital, Lecce, Italy; 8 Medical Genetics Unit, St. Bassiano Hospital, Bassano del Grappa, Italy; 9 Dipartimento Pediatria, Genetica Clinica, Padova, Italy; 10 Department of Genetics, Institute of Child Health, "Aghia Sophia" Children's Hospital, Athens, Greece; 11 Dipartimento Materno Infantile, Università degli studi Modena, Italy; 12 U.O. Malattie Metaboliche PO Giovanni XXIII, AOU Policlinico Consorziale, Bari, Italy; 13 Clinical Genetics Unit, S.Maria Nuova Hospital Reggio Emilia, Italy; 14 Medical Genetics Unit, Children's Hospital Anna Meyer, Firenze, Italy; 15 Genetica Medica, Università di Catania, Catania, Italy; 16 Area Funzionale di Genetica Clinica Pediatrica, Dipartimento di Pediatria, Università degli Studi di Napoli "Federico II", Italy; 17 Biologia Molecolare e Citogenetica, Diagnostica e Ricerca San Raffaele, Milano, Italy; 18 Unita' Operativa di Genetica Medica, Azienda Ospedaliera Bianchi-Melacrino-Morelli, Reggio Calabria, Italy; 19 Medical Genetics Unit, University of Perugia, "S. Maria della Misericordia" Hospital, Perugia, Italy; 20 Medical Genetics Section, Biotechnology Department, University of Siena, Italy. UOC Genetica Medica, Dipartimento di Emergenza Urgenza e dei Servizi Diagnostici, Azienda Ospedaliera Universitaria Senese, Siena, Italy; 21 UOC Genetica Medica, Azienda Ospedaliera RN "G.Rummo", Benevento, Italy; 22 Medical Genetics Section, Cytogenetics and Molecular Genetics Unit, Santa Chiara University Hospital, Pisa, Italy; 23 Ambulatorio di Genetica Medica, Azienda ULSS 9, Treviso, Italy; 24 Newborn Intensive Care Unit, Maggiore Hospital, Bologna, Italy; 25 Servizio di Genetica, ASL NAPOLI 1 P.S.I. Elena d'Aosta Napoli, Italy; 26 Stituto di Scienze Materno-Infantili, Università Politecnica delle Marche, Ancona, Italy

## Abstract

**Background:**

Kabuki syndrome (Niikawa-Kuroki syndrome) is a rare, multiple congenital anomalies/mental retardation syndrome characterized by a peculiar face, short stature, skeletal, visceral and dermatoglyphic abnormalities, cardiac anomalies, and immunological defects. Recently mutations in the histone methyl transferase *MLL2 *gene have been identified as its underlying cause.

**Methods:**

Genomic DNAs were extracted from 62 index patients clinically diagnosed as affected by Kabuki syndrome. Sanger sequencing was performed to analyze the whole coding region of the *MLL2 *gene including intron-exon junctions. The putative causal and possible functional effect of each nucleotide variant identified was estimated by *in silico *prediction tools.

**Results:**

We identified 45 patients with *MLL2 *nucleotide variants. 38 out of the 42 variants were never described before. Consistently with previous reports, the majority are nonsense or frameshift mutations predicted to generate a truncated polypeptide. We also identified 3 indel, 7 missense and 3 splice site.

**Conclusions:**

This study emphasizes the relevance of mutational screening of the *MLL2 *gene among patients diagnosed with Kabuki syndrome. The identification of a large spectrum of *MLL2 *mutations possibly offers the opportunity to improve the actual knowledge on the clinical basis of this multiple congenital anomalies/mental retardation syndrome, design functional studies to understand the molecular mechanisms underlying this disease, establish genotype-phenotype correlations and improve clinical management.

## Background

Kabuki syndrome (KS, MIM #147920), also known as Niikawa-Kuroki syndrome, is a rare, multiple congenital anomalies/mental retardation syndrome characterized by a peculiar face, which is defined by long palpebral fissures with eversion of the lateral third of the lower eyelids, short columella with a broad and depressed nasal tip, prominent ears, and a cleft or high-arched palate. Additional features include short stature, skeletal, visceral and dermatoglyphic abnormalities, cardiac anomalies, and immunological defects [1,2]. Kabuki syndrome has an incidence of 1 in 32,000, likely largely underestimated [3]. The vast majority of reported cases are sporadic. After initial and controversial data that associated this condition to chromosomal rearrangement [4,5], mutations in the *MLL2 *gene identified the underlying cause of Kabuki syndrome in approximately 72% of affected individuals [6,7]. The encoded MLL2 protein is a member of the Mixed Lineage Leukemia (MLL) family of histone methyl transferases (HMT). The MLL proteins (MLLs) are part of the SET (Su(var)3-9, Enhancer-of-zeste, Trithorax) family of proteins [8]. The highly conserved SET domain of MLLs confers histone methyltransferase activity, which is the core function of HMTs. MLLs are important in the epigenetic control of active chromatin states [9]. They act as transcriptional co-activators and are involved in embryogenesis and development through, for example, regulation of the expression of the *HOX *genes and their interaction with nuclear receptors [10,11].

The *MLL2 *gene encodes a multi-domain-containing protein of 5,537 amino acid residues that can methylate the Lys-4 position of histone H3 (H3K4), an epigenetic mark correlated with transcriptional active chromatin [12,13]. MLL2 is involved in estrogen receptor α (ERα)-mediated signal transduction, acting as a coactivator of a complex that includes ASH2, RBQ3, and WDR5 [14].

In the present study, by direct sequencing of DNA samples from 62 Kabuki patients we identified 42 *MLL2 *variants, 38 of which are novel.

## Methods

### Subjects and Clinical Data

**Figure 1:**
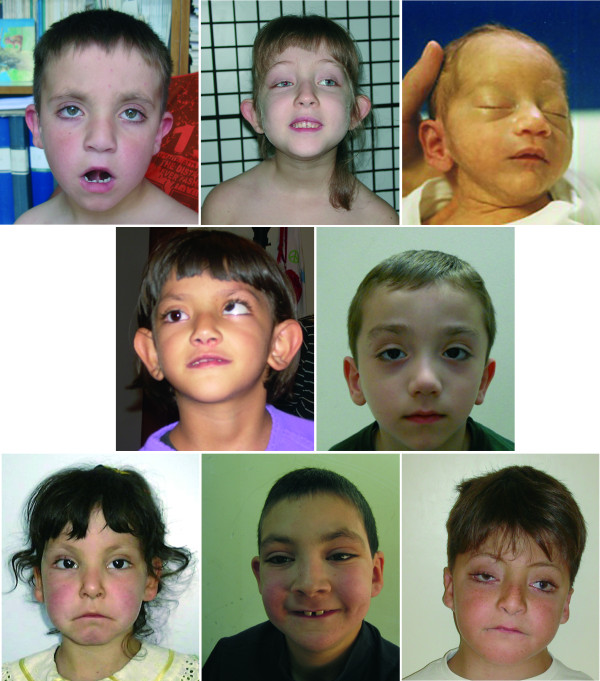
**Facial features of Kabuki syndrome patients**. Representative images of Kabuki patients with *MLL2 *mutations.

Our cohort comprised 62 index patients clinically diagnosed as affected by Kabuki syndrome (Figure 1 and Table 1). Patients were enrolled after obtaining appropriate informed consent by the physicians in charge and approval by the respective local ethics committees. Patients were included in this study whether at least four of the following inclusion criteria were present: 1) long palpebral fissures with eversion of the lateral portion of lower eyelid; 2) broad, arched eyebrows with sparseness; 3) short nasal columella with depressed nasal tip; 4) large, prominent or cupped ears; 5) developmental delay-mental retardation [15].

**Table 1:** Clinical features of our cohort of Kabuki syndrome patients

**Gender**	36/62 (58.1%) Male
	26/62 (41.9) Female
**General features**	
Short stature	38/62 (61.2)
Microcephaly	18/62 (29)
Neonatal problems	42/62 (67.7)
**Facial**	
Long palpebral fissures	59/62 (95.2)
Everted lower eyelids	53/62 (85.5)
Large dysplastic ears	56/62 (90.3)
Arched eyebrows, sparse lateral one third	51/62 (82.2)
Flat nasal tip	43/62 (69.3)
Abnormal dentition	33/62 (53.2)
High/cleft palate	37/62 (59.7)
Strabismus	26/62 (41.9)
Blue sclerae	11/62 (17.7)
Micrognathia	20/62 (32.2)
Ptosis	32/62 (51.6)
Broad nasal root	39/62 (62.9)
Oligodontie	23/62 (37.1)
Thin upper and full lower lip	44/62 (71)
**Limb/skeletal**	
Persistent fetal pads	47/62 (75.8)
Brachy/clinodactyly	39/62 (62.9)
Lax joints	30/62 (48.4)
Hip dislocation	8/62 (12.9)
**Visceral anomalies**	
Cardiac anomalies	37/62 (59.7)
Urogenital anomalies	24/62 (38.7)
**Neurologic**	
MR	52/62 (83.9)
Hypotonia	37/62 (59.7)
Seizures	13/62 (21.4)
**Other clinical features (most recurrent)**	
frequent infections	26/62 (41.9)
leftearly breast development	10/62 (16.1)
lefthypoacusia	7/62 (11.3)
skeletal anomalies	6/62 (9.7)
leftthyroid anomalies	4/62 (6.9)
leftagenesis/dysgenesis corpus callosum	3/62 (5.2)

### Samples preparation

Genomic DNAs were extracted from fresh and/or frozen peripheral blood leukocytes of the probands and their available family members using an automated DNA extractor and commercial DNA extraction Kits (EZ1, Qiagen, Hilden, Germany).

### PCR-based sequencing of MLL2

Primers were designed using the Primer 3 Output program (http://frodo.wi.mit.edu/primer3/) to amplify the 54 coding exons of *MLL2 *(RefSeq NM_003482.3) gene including the intronic flanking sequences. Amplicons and primers were checked both by BLAST and BLAT against the human genome to ensure specificity. A complete list of primers is reported in Additional file 1, Table S1. The amplified products were subsequently purified and sequenced with a ready reaction kit (BigDye Terminator v1.1 Cycle, Applied Biosystems). The fragments obtained were purified using DyeEx plates (Qiagen) and resolved on an automated sequencer (3130xl Genetyc analyzer DNA Analyzer, ABI Prism). Sequences were analyzed using the Sequencer software (Gene Codes, Ann Arbor, Michigan). Whenever possible the mutations identified were confirmed on a second independent blood sample. The issue of whether the novel *MLL2 *missense alterations were causative mutations or neutral polymorphisms was addressed by searching dbSNP (http://www.ncbi.nlm.nih.gov/SNP) for their presence; the screening of 100 alleles from healthy unrelated control subjects and from the 1000 Genomes database [16] were used to assess their presence/absence in the general population. All existing and new mutations were described following the recommendations of the Human Genome Variation Society (http://www.hgvs.org/mutnomen).

### In silico analysis

The putative causal and functional effect of each identified nucleotide variant was estimated by using the following *in silico *prediction tools: Polyphen http://genetics.bwh.harvard.edu/pph, Align GVGD http://agvgd.iarc.fr/agvgd_input.php, and MutPred http://mutdb.org/profile. Splice sites variants were evaluated for putative alteration of regulatory process at the transcriptional or splicing level with NetGene2 http://www.cbs.dtu.dk/services/NetGene2 and NNSPLICE http://www.fruitfly.org/seq_tools/splice.html. RESCUE-ESE http://genes.mit.edu/burgelab/rescue-ese/ and Fas-ESS http://genes.mit.edu/fas-ess/ online tools were used to predict exon-splicing enhancer and silencer, respectively. RepeatMasker http://www.repeatmasker.org/ was used to screen DNA sequences for the presence of direct repeats. The Coils program http://www.ch.embnet.org/software/COILS_form.html was employed to calculate the probability that the variant induces a conformational change in the coiled-coil domains. Finally multiple species alignment of MLL2 protein was made with the ClustalW software http://www.ebi.ac.uk/Tools/clustalw2/index.html using the following orthologs sequences obtained through the Ensembl genome browser http://www.ensembl.org: *P. troglodytes *(ENSPTRP00000041051), *M. musculus *(ENSMUSP00000023741), *C. familiaris *(gENSCAFP00000012833), *B. taurus *(ENSBTAP00000019193), *X. troplicalis *(ENSXETP00000024427), and *D. rerio *(ENSDARP00000053862).

## Results and Discussion

**Figure 2:**
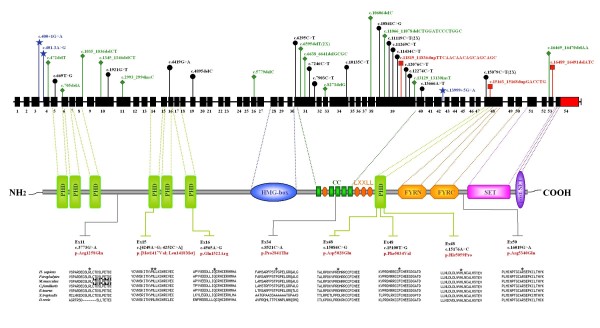
**Mutation spectrum of *MLL2 *in our cohort of Kabuki syndrome patients**. Upper panel: genomic structure of the *MLL2 *gene including 54 coding exons (black rectangles), the 3'untranslated region (red rectangle) and introns (black horizontal line). Mutations are represented in black (nonsense = 16), red (indel = 3), blue (splice sites = 3), and green (frameshift = 13). Middle figure: MLL2 protein domain structures, PHD, plant homeodomain finger; HMG-box, high mobility group; CC, Coiled Coil; LXXLL domain, FYRN, FY-rich domain, N-terminal region; FYRC, FY-rich domain, C-terminal region; SET, (Su(var)3-9, Enhancer-of-zeste, Trithorax) domain; PostSET: PostSET domain. Lower panel: evolutionary comparison of the protein sequences flanking the 7 missense mutations identified in the human MLL2 protein with their orthologous counterparts in seven species. The position of the amino acid change is indicated with a star.

Exome sequencing recently revealed that mutations in the histone methyltransferase *MLL2 *gene are a major cause of Kabuki syndrome [6]. In a collaborative effort that involved Italian Institutes, except one, we enrolled 62 individuals with a clinical diagnosis of sporadic Kabuki syndrome (Table 1). We detected nucleotide variants in 73% of the patients (45/62) by direct sequencing of all 54 exons of the *MLL2 *gene; the vast majority of which are novel (90%, 38/42 different variants) (Figure 2, Additional file 1, Table S2, Additional file 2, Figure S1 and below) [6,7].

### Nonsense and frameshift mutations

In agreement with previous reports we identified a majority of truncating mutations (70%, 29/42), three of which were reported previously (Figure 2, Additional file 1, Table S2) [6,7]. Most of the variants are predicted, if translated, to encode shorter MLL2 proteins either by loss of the entire C-terminal region or parts of it (Figure 2). This region harbors highly conserved domains that are found in a variety of chromatin-associated proteins [17-19]: (i) the helical LXXLL regions involved in the recruitment of the MLL2 complex to the promoters of ERα target genes (Figure 2); (ii) FYRN and FYRC sequence motifs, two poorly characterized phenylalanine/tyrosine-rich regions of around 50 and 100 amino acids [20], respectively; and (iii) a catalytic SET motif that confers histone methyltransferases activity.

Although it has not been yet experimentally verified for the *MLL2 *gene, the prevalence of premature termination mutations may result in the partial transcripts degradation through nonsense-mediated mRNA decay (NMD). NMD is an evolutionarily conserved process that typically degrades transcripts containing premature termination codons (PTCs) to prevent translation of unnecessary or aberrant and possible transcripts [21]. The NMD process takes place when PTCs are located more than 50-55 nucleotides upstream of an exon-exon junction [22]. As 86% (25/29) of such detected *MLL2 *mutations follow this rule it is likely that the consequent MLL2 haploinsufficiency could be the driving force for the onset of the Kabuki syndrome.

### Indel variants

Our screen revealed three not yet described indel variants located in the C-terminal region of the protein (see samples KB71, KB77, and KB53 in Additional file 1, Table S2). They might have resulted from slipped mispairing between direct repeats or through the insertion or deletion of a single base within a mononucleotide tract (Additional file 1, Table S3), as already reported [23].

COILS algorithm predicted the amplification of one of the five coiled-coil putative domains for the c.11819_11836dupTTCAACAACAGCAGCAGC (p.Lys3940_Gln3945dup) (Figure 2 and data not shown), a domain involved in protein-protein interaction. This variant was inherited from the apparently asymptomatic mother. It is thus impossible to conclusively determine the pathogenic nature of the resulting protein.

### Splice site variants

We detected 3 variants located at the splice site junctions, two of which are novel [7] (Additional file 1, Table S2); the *in silico *modeling predicted complete or partial abrogation of the junction formation with a pathogenic impact. The c.400+1G>A, occurring within the invariant GT donor splice site in intron 3-4, results in the disruption of the canonical splice site and it is expected to produce an aberrant protein of only 135 residues. The c.401-3A>G, occurring 3 bp away from the next intron-exon junction, is predicted to create a new acceptor splice site at position -3 within intron 3-4 that could lead to a frameshift encoding a mutant protein with a premature stop at 84 codons downstream. Finally, c.13999+5G>A decreases the donor site score prediction, possibly resulting in a less efficient intron splicing. Unfortunately, RNA from these patients was unavailable preventing further investigation of the effect of these variants.

### Missense variants

Missense *de novo *variants have already been found in Kabuki patients. Ng and colleagues reported 8 pathogenic missense variants, two of which were recurrent in affected patients. As these were all mapping within the last exon of *MLL2 *that encodes the different conserved C-terminal domains of the protein (see above), the authors suggested that such mutations are tolerated, while mutations elsewhere are lethal. By *in silico *analysis, Paulussen *et al*. proposed the pathogenicity of two missense variants located within that C-terminal region [6,7]. We detected seven patients with a single or two missense variants (KB28 and KB38 patients; Additional file 1, Table S2). PCR amplification, cloning and sequencing showed that both sets of the two sequence changes in KB28 and KB38 patients are located on one allele. From a phenotypic point of view, the two patients with pairs of missense variants do not appear to be more severely affected than affected individuals with single variants. Sequencing of the corresponding exons in the KB38 parents demonstrated that both variants arose *de novo*, while the KB28 patient inherited the variant from the apparently asymptomatic mother. We had also accessed to DNA of parents of carriers of missense variants (Additional file 1, Table S2). Yet, in both cases the *MLL2 *variant was inherited from the apparently asymptomatic father.

The missense variants are distributed across the entire length of the *MLL2 *gene (Figure 2). They were not found in 50 healthy unrelated control samples and were absent from the 1000 Genomes database [16]. The putative functional relevance and pathogenicity of these *MLL2 *missense variants were predicted by *in silico *software. The PolyPhen program, which predicts possible impact of an amino acid substitution on the structure and function of a human protein, identified only the p.Pro2841Thr variant as possibly damaging. Accordingly to the criteria of Align-GVGD all missense variants were predicted to be deleterious (Table 2). Finally, we used the computational model MutPred, designed to classify an amino acid substitution as disease-associated or neutral in human. MutPred predicted that four of the identified missense variants have a high probability (≥0,5) of being deleterious and generated *in silico *hypothesis for the possible pathological mechanism for three of them (Table 2). The analysis of the mutated residues in 7 MLL2 proteins orthologs showed that.all the missense variants occurred at amino acid residues evolutionarily conserved (Figure 2).

**Table 2:** In *silico *prediction of pathogenic effect of *MLL2 *missense and splice site variants

				Prediction of damaging effect at the protein level					
						
ID	Exon	Mutation	AA change			MutPred		Rescue-ESE	Fas-ESS
								
				Polyphen	Align GVGD	Probability of deleterious mutation	Confident *in silico *hypothesis		
KB32	11	c.3773G>A	p.Arg1258Gln	benign	deleterious	0.16	loss of loop (p = 0.0288, loss of catalytic residue at R1258 (p = 0.0301); gain of helix (p = 0.0349)	no change	no change
KB28	15	c.[4249A>G;4252C>A]	p.[Met1471Val;Leu1418Met]	benign; benign	deleterious; deleterious	0.46; 0.47	none; none	no change	gain of one ESS
KB34	16	c.4565A>G	p.Gln1522Arg	benign	deleterious	0.50	none	loss of one ESE	no change
KB27	34	c.8521C>A	p.Pro2841Thr	possibly damaging	deleterious	0,24	gain of phoshorylation P2841 (p = 0.028)	gain of two ESEs	loss of one ESS
KB38	48	c.[15084C>G;15100T>G]	p.[Asp 5028Glu;Phe5034Val]	benign; benign	deleterious; deleterious	0.42; 0.56	none; none	gain of three ESEs	loss of three ESSs
KB76	48	c.1517A>C	p.His5059pro	benign	deleterious	0,71	none	no change	no change
KB17	50	c.16019G>A	p.Arg5340Gln	benign	deleterious	0,53	gain of ubiquitination at K5344 (p = 0.0396)	gain of two ESEs	no change

**ID**		**Intron**	**Mutation**	**Splice site modification prediction**		**Predicted Protein**	**Conclusion**		
								
				**NetGene**	**NNSplice**				

KB31		Intron 3-4	c.400+1G>A	Loss of DS	Loss of DS	135 AA with novel 2 AA	deleterious		
KB20		Intron 3-4	c.401-3A>G	New AS at-3	New AS at-3	217 AA with novel 84 AA	deleterious		
KB29		Intron 42-43	c. 13999+5 G>A	Lower confidence of DS prediction	Lower confidence of DS prediction		unpredictable		

Finally, we employed RESCUE-ESE and Fas-ESS tools on missense variants and frameshift mutations to predict associated splicing phenotypes by identifying sequence changes that disrupt or alter predicted Exonic Splicing Enhancers (ESE) and Exonic Splicing Silencers (ESS). ESE and ESS are short oligonucleotides that can enhance or inhibit pre-mRNA splicing when present in exons, playing important roles in constitutive and alternative splicing. A variation that disrupts an ESE, for instance, could cause exon skipping which would result in the exclusion of an entire exon from the mRNA transcript. Conversely, a substitution in the ESS sequence promotes the use of adjacent splice sites, often contributing to alternative splicing. As reported in Table 2 and Additional file 1, Table S3, we found that some of the *MLL2 *mutations lead to creation of new ESEs and/or to disruption of predicted wild type ESEs/ESSs. As secondary structures or adjacent negative elements also participate to the modulation of the splicing event mediated by ESE and ESS, we retain that association to functional studies will enable to better understand the role of the reported cases of ESEs/ESSs disruption or alteration in the complex phenotypic spectrum observed in the Kabuki patients.

## Conclusions

Our study increases the number of identified *MLL2 *mutations and variants, and emphasizes some characteristics of the spectrum of *MLL2 *mutations associated with this pathology, further providing insight into its etiology. The *in silico *analysis predicts that the identified *MLL2 *missense, splice-site and indel variants might be pathogenic. Other studies reported the presence of such *MLL2 *variants predicted to be associated with the disease. However, their biological significance and pathogenicity were not unambiguously demonstrated; therefore further and more functionally oriented studies are needed to understand the nature of these variants and their possible role in the disease. Solving these issues is relevant to avoid any incorrect interpretation and diagnosis of other Kabuki cases carrying such *MLL2 *variants.

We were unable to found any detectable point mutation and/or small del/dup in the coding region of *MLL2 *gene in 27% (17/62) of the Kabuki syndrome patients. Mutations in *MLL2 *regulatory regions, exon microduplications and/or microdeletions, as well as genetic heterogeneity of the syndrome may account for these negative results. Alternatively, some of these patients might have been misdiagnosed as a result of the complex clinical spectrum covered by this pathology, thus possibly highlighting the need to more accurately select Kabuki cases before conducting the analysis.

In summary, this study underlines the relevance of mutational screening of the *MLL2 *gene among patients with Kabuki syndrome. The identification of a large spectrum of *MLL2 *mutations will offer the opportunity to improve the actual knowledge on the clinical basis of this multiple congenital mental retardation syndrome, to design functional studies to understand the molecular mechanisms underlying the disease, to establish genotype-phenotype correlations, to improve the clinical management, and to identify potential targets for therapy.

## Competing interests

The authors declare that they have no competing interests.

## Authors' contributions

GM designed the study and obtained the necessary financial support. BA, MNL, CF, AC, and EVD carried out the molecular genetic studies. LM and GM interpreted the results and wrote the manuscript with the help of LZ and AR. All other authors provided samples. All authors read and approved the final manuscript.

## Supplementary Material

Additional file 1 **Table S1**. Oligos used in this study. **Table S2**. *MLL2 *mutations identified in our cohort of KS patients and as reported in the literature. **Table S3**. Repeats (underlined and highlighted in red) that might mediate micro-deletions, micro-insertion/deletions (indel), and micro-duplications in the *MLL2 *gene. Click here for file

Additional file 2 **Figure S2**. Frequency of different *MLL2 *mutation types in Kabuki syndrome patients identified to date. Our study (A), Ng *et al*. and Paulussen *et al*. studies (B), all three studies, A+B, (C). Click here for file

## References

[B1] Kuroki Y Suzuki Y Chyo H Hata A Matsui I A new malformation syndrome of long palpebral fissures, large ears, depressed nasal tip, and skeletal anomalies associated with postnatal dwarfism and mental retardation J Pediatr 1981 99 4 570 573 10.1016/S0022-3476(81)80256-9 7277097

[B2] Niikawa N Matsuura N Fukushima Y Ohsawa T Kajii T Kabuki make-up syndrome: a syndrome of mental retardation, unusual facies, large and protruding ears, and postnatal growth deficiency J Pediatr 1981 99 4 565 569 10.1016/S0022-3476(81)80255-7 7277096

[B3] Niikawa N Kuroki Y Kajii T Matsuura N Ishikiriyama S Tonoki H Ishikawa N Yamada Y Fujita M Umemoto H Kabuki make-up (Niikawa-Kuroki) syndrome: a study of 62 patients Am J Med Genet 1988 31 3 565 589 10.1002/ajmg.1320310312 3067577

[B4] Cusco I del Campo M Vilardell M Gonzalez E Gener B Galan E Toledo L Perez-Jurado LA Array-CGH in patients with Kabuki-like phenotype: identification of two patients with complex rearrangements including 2q37 deletions and no other recurrent aberration BMC Med Genet 2008 9 27 18405349 10.1186/1471-2350-9-27 PMC2358878

[B5] Milunsky JM Huang XL Unmasking Kabuki syndrome: chromosome 8p22-8p23.1 duplication revealed by comparative genomic hybridization and BAC-FISH Clin Genet 2003 64 6 509 516 10.1046/j.1399-0004.2003.00189.x 14986831

[B6] Ng SB Bigham AW Buckingham KJ Hannibal MC McMillin MJ Gildersleeve HI Beck AE Tabor HK Cooper GM Mefford HC Lee C Turner EH Smith JD Rieder MJ Yoshiura K Matsumoto N Ohta T Niikawa N Nickerson DA Bamshad MJ Shendure J Exome sequencing identifies MLL2 mutations as a cause of Kabuki syndrome Nat Genet 2010 42 9 790 793 10.1038/ng.646 20711175 PMC2930028

[B7] Paulussen AD Stegmann AP Blok MJ Tserpelis D Posma-Velter C Detisch Y Smeets EE Wagemans A Schrander JJ van den Boogaard MJ van der Smagt J van Haeringen A Stolte-Dijkstra I Kerstjens-Frederikse WS Mancini GM Wessels MW Hennekam RC Vreeburg M Geraedts J de Ravel T Fryns JP Smeets HJ Devriendt K Schrander-Stumpel CT MLL2 mutation spectrum in 45 patients with Kabuki syndrome Hum Mutat 2010 10.1002/humu.21416 21280141

[B8] Dillon SC Zhang X Trievel RC Cheng X The SET-domain protein superfamily: protein lysine methyltransferases Genome Biol 2005 6 8 227 10.1186/gb-2005-6-8-227 16086857 PMC1273623

[B9] Issaeva I Zonis Y Rozovskaia T Orlovsky K Croce CM Nakamura T Mazo A Eisenbach L Canaani E Knockdown of ALR (MLL2) reveals ALR target genes and leads to alterations in cell adhesion and growth Mol Cell Biol 2007 27 5 1889 1903 10.1128/MCB.01506-06 17178841 PMC1820476

[B10] Ansari KI Mandal SS Mixed lineage leukemia: roles in gene expression, hormone signaling and mRNA processing FEBS J 2010 277 8 1790 1804 10.1111/j.1742-4658.2010.07606.x 20236313

[B11] Eissenberg JC Shilatifard A Histone H3 lysine 4 (H3K4) methylation in development and differentiation Dev Biol 2010 339 2 240 249 10.1016/j.ydbio.2009.08.017 19703438 PMC3711867

[B12] Malik S Bhaumik SR Mixed lineage leukemia: histone H3 lysine 4 methyltransferases from yeast to human FEBS J 2010 277 8 1805 1821 10.1111/j.1742-4658.2010.07607.x 20236312 PMC2873088

[B13] Strahl BD Ohba R Cook RG Allis CD Methylation of histone H3 at lysine 4 is highly conserved and correlates with transcriptionally active nuclei in Tetrahymena Proc Natl Acad Sci USA 1999 96 26 14967 14972 10.1073/pnas.96.26.14967 10611321 PMC24756

[B14] Mo R Rao SM Zhu YJ Identification of the MLL2 complex as a coactivator for estrogen receptor alpha J Biol Chem 2006 281 23 15714 15720 10.1074/jbc.M513245200 16603732

[B15] Kawame H Hannibal MC Hudgins L Pagon RA Phenotypic spectrum and management issues in Kabuki syndrome J Pediatr 1999 134 4 480 485 10.1016/S0022-3476(99)70207-6 10190924

[B16] Durbin RM Abecasis GR Altshuler DL Auton A Brooks LD Gibbs RA Hurles ME McVean GA A map of human genome variation from population-scale sequencing Nature 2010 467 7319 1061 1073 10.1038/nature09534 20981092 PMC3042601

[B17] Balciunas D Ronne H Evidence of domain swapping within the jumonji family of transcription factors Trends Biochem Sci 2000 25 6 274 276 10.1016/S0968-0004(00)01593-0 10838566

[B18] Doerks T Copley RR Schultz J Ponting CP Bork P Systematic identification of novel protein domain families associated with nuclear functions Genome Res 2002 12 1 47 56 10.1101/gr.203201 11779830 PMC155265

[B19] Prasad R Zhadanov AB Sedkov Y Bullrich F Druck T Rallapalli R Yano T Alder H Croce CM Huebner K Mazo A Canaani E Structure and expression pattern of human ALR, a novel gene with strong homology to ALL-1 involved in acute leukemia and to Drosophila trithorax Oncogene 1997 15 5 549 560 10.1038/sj.onc.1201211 9247308

[B20] Garcia-Alai MM Allen MD Joerger AC Bycroft M The structure of the FYR domain of transforming growth factor beta regulator 1 Protein Sci 2010 19 7 1432 1438 10.1002/pro.404 20506279 PMC2970912

[B21] Maquat LE When cells stop making sense: effects of nonsense codons on RNA metabolism in vertebrate cells RNA 1995 1 5 453 465 7489507 PMC1482424

[B22] Nagy E Maquat LE A rule for termination-codon position within intron-containing genes: when nonsense affects RNA abundance Trends Biochem Sci 1998 23 6 198 199 10.1016/S0968-0004(98)01208-0 9644970

[B23] Tappino B Biancheri R Mort M Regis S Corsolini F Rossi A Stroppiano M Lualdi S Fiumara A Bembi B Di Rocco M Cooper DN Filocamo M Identification and characterization of 15 novel GALC gene mutations causing Krabbe disease Hum Mutat 2010 31 12 E1894 1914 10.1002/humu.21367 20886637 PMC3052420

[B24] Ensembl Genome Browser http://www.ensembl.org

