# Supplementary material for: Mutation spectrum of MLL2 in a cohort of kabuki syndrome patients
Source: Orphanet J Rare Dis. 2011 Jun 9;6:38. doi: 10.1186/1750-1172-6-38 (PMC3141365; doi:10.1186/1750-1172-6-38)
Supplement: Additional file 1 — Table S1. Oligos used in this study. Table S2. MLL2 mutations identified in our cohort of KS patients and as reported in the literature. Table S3. Repeats (underlined and highlighted in red) that might mediate micro-deletions, micro-insertion/deletions (indel), and micro-duplications in the MLL2 gene. [file 1750-1172-6-38-S1.DOC]

**Table S1.** Oligos used in this study

| **Name** | **Sequence** | **Type** | **Amplified Exons** | **Amplicon Size** |
| --- | --- | --- | --- | --- |
| MLL2_EX 1F | TCTTTCTTGGCACCAACAGG | PCR | 1 to 4 | 1155 |
| MLL2_EX 2R | TTCCCCAACACTCATTTTCC | Sequence |
| MLL2_EX 4R | TGGTCCTTCTCATTCCAACC | PCR/Sequence |
| MLL2_EX 5F | TGGGCTTCTGAGAGTCAGGT | PCR/Sequence | 5 | 340 |
| MLL2_EX 5R | CCAAGGCACATTTGGTCTCT | PCR |
| MLL2_EX 6F | CATGGAGTGCCTGTTTTGTG | PCR/Sequence | 6 to 9 | 1230 |
| MLL2_EX 9R | TGCACAAACTGTCTCTTGCC | PCR/Sequence |
| MLL2_EX 10_1F | CAGACCCCACACTTTCTGTG | PCR/Sequence | 10 | 1713 |
| MLL2_EX 10_2F | AATCGCCCCTCTCTCCG | Sequence |
| MLL2_EX 10_3F | CACCTGAGGACTCACCTGC | Sequence |
| MLL2_EX 10_3R | GGAGGAAGGGGATCTGGAAG | PCR |
| MLL2_EX 11_1F | CAGCTGTAACTCTTCATGGCAC | PCR/Sequence | 11 | 1269 |
| MLL2_EX 11_2F | CAGCCTTGGAACCCAGTG | Sequence |
| MLL2_EX 11_2R | GATTCACAAAGCAAGGTGGG | PCR |
| MLL2_EX 12F | ACCCAACACACTAGCCCTTG | PCR/Sequence | 12 to 14 | 1424 |
| MLL2_EX 13R | CCCATCTATCCTCTCACCAAAC | Sequence |
| MLL2_EX 14F | TGACTCTGGTCGCAAATCAG | Sequence |
| MLL2_EX 14R | ATGAACAACCATGACCGATG | PCR |
| MLL2_EX 15F | TTGTACCTGAGAGGCAGAGG | PCR | 15 to 18 | 1273 |
| MLL2_EX 16R | CCACATCGTCCTCTGTGAAG | Sequence |
| MLL2_EX 17F | GTGAACGGTGAGAACATCCC | Sequence |
| MLL2_EX 18R | CGAAATCCTAGCAGTGAAGAGAC | PCR |
| MLL2_EX 19F | GCTGGACTTTTGTCACTGCC | PCR | 19 to 22 | 1290 |
| MLL2_EX 20R | GTGGATGAGAAGCCGCTG | Sequence |
| MLL2_EX 22R | GGGCAGTCAAGGAGAAACAG | PCR/Sequence |
| MLL2_EX 23F | AGCAGTGAGGGAGGTGTCTG | PCR | 23 to 27 | 1352 |
| MLL2_EX 24R | GCACAGCAGAGGGACAGAG | Sequence |
| MLL2_EX 25F | ATGGCCCTCTGTGGTTCAG | Sequence |
| MLL2_EX 27R | GAACTCAGATGGAGGGAAAGG | PCR |
| MLL2_EX 28F | TCTAGGCAGTTTGTCTGGGG | PCR | 28 to 30 | 900 |
| MLL2_EX 29R | TACCCAAAGATCCCTCCCTC | Sequence |
| MLL2_EX 30F | ATGGATTAGCGTGGGAACTG | Sequence |
| MLL2_EX 30R | CACCTTGGTCTGCTTGTTGA | PCR |
| MLL2_EX 31_1F | CGCATCAACAAGGTGCAG | PCR/Sequence | 31-a | 1037 |
| MLL2_EX 31_2F | CCTGCCTATCCCCTGGAG | Sequence |
| MLL2_EX 31_2R | TGGGTCAGTGTAGGAGCCAG | PCR |
| MLL2_EX 31_3F | CATCTCAGGTAGAGCCCCAG | PCR/Sequence | 31-b | 1103 |
| MLL2_EX 31_4R | ATAGGGCTGCCCCAGAGAC | PCR/Sequence |
| MLL2_EX 32F | ATATCGCTCCTGTCTCTGGG | PCR/Sequence | 32 to 33 | 530 |
| MLL2_EX 33R | AACAGTGATAAAATCCATCCCC | PCR |
| MLL2_EX 34_1F | TAAGCCCATGTTCTTGCTCC | PCR | 34 | 1996 |
| MLL2_EX 34_1R | ACACCCAGACCCAGGTGAG | Sequence |
| MLL2_EX 34_2F | CTGGAAGCTGGGAAGTTGC | Sequence |
| MLL2_EX 34_3R | TCAGTGCCCATTTAGGGATAAC | PCR/Sequence |
| MLL2_EX 35F | CAGGATGTTGAAGGGAATCG | PCR | 35 to 38 | 1004 |
| MLL2_Ex 37R | TCCTCCATATGACCCAAACC | Sequence |
| MLL2_EX 38F | TGGTTTGGGTCATATGGAGG | Sequence |
| MLL2_EX 38R | ATGCCAACCCTCTTCCCTG | PCR |
| MLL2_EX 39_1F | AGCAGCAGAGCAAGATCCAG | PCR/Sequence | 39-a | 1035 |
| MLL2_Ex 39_2R | GACTCTGCTGAAGATGGGACA | PCR |
| MLL2_EX 39_2F | GAAGCCTCGGACCTGATTC | PCR/Sequence | 39-b | 1084 |
| MLL2_EX 39_3F | AGGTGCTTATGACCCAGTCC | Sequence |
| MLL2_EX 39_3R | GAGGGCTTTACCTCTCCTG | PCR |
| MLL2_EX 39_4F | CAGATGGGCCTTTTAAACCA | PCR/Sequence | 39-c | 1748 |
| MLL2_EX 39_5F | CAGTGGATCATCTTCTGAGGC | Sequence |
| MLL2_Ex39_6_F | TCCCAGCTTCCCACTGAG | Sequence |
| MLL2_EX 39_6R | GCCAAATAAGCCCATTGAAG | PCR/Sequence |
| MLL2_EX 40F | GAGCCTGGGTCAGACAGAAG | PCR/Sequence | 40 to 42 | 960 |
| MLL2_EX 42R | CCTCAGGTGCCCTGTTATGT | PCR/Sequence |
| MLL2_EX 43F | CAAACTGGTAGGTGGGAGGA | PCR/Sequence | 43 to 45 | 919 |
| MLL2_EX 45R | TCTAGCCCAGGCTTTCACAT | PCR/Sequence |
| MLL2_EX 46F | CTAGGTGTCCTTGTCCCCAC | PCR/Sequence | 46 to 47 | 530 |
| MLL2_EX47R | CTTGCCTCCCAAAGCACTG | PCR |
| MLL2_EX 48_1F | AAGAATGTGGAGGCCTTTTG | PCR/Sequence | 48 | 1428 |
| MLL2_EX 48_2R | AGGGTGACAAGAGAGGCTCA | PCR/Sequence |
| MLL2_EX 49F | GCCAAGAGAGGATCTGGAAG | PCR | 49 to 50 | 634 |
| MLL2_EX 50R | TGATTCCCCATTTTCTCCAC | PCR/Sequence |
| MLL2_EX 51F | CAGAGGAGGTGGGTGGTATG | PCR | 51 to 54 | 1522 |
| MLL2_EX 51R | TTCTCCTGCCTTTCCCTTCT | Sequence |
| MLL2_EX 52F | AGAGCAGGAGGAAAGCTGTG | Sequence |
| MLL2_EX 54R | CGGCCACACATCCTCTTC | PCR/Sequence |

**Table S2.** *MLL2* mutations identified in our cohort of KS patients and as reported in the literature

| **Mutation**  **type** | **ID** | **Exon/**  **Intron** | **Mutation** | **AA change** | **Inheritance** | **Reference** |
| --- | --- | --- | --- | --- | --- | --- |
| **nonsense** | **KB49** | **5** | **c.669T>G** | **p.Tyr223X** | **NA** | **This study** |
|  | **KB35** | **10** | **c.1921G>T** | **p.Glu641X** | **NA** | **This study** |
|  | **KB33** | **16** | **c.4419G>A** | **p.Trp1473X** | **NA** | **This study** |
|  |  | 19 | c.4843C>T | p.Arg1615X | NA | Ng et al. 2010 |
|  | **KB63** | **19** | **c.4895delC** | **p.Ser1632X** | **NA** | **This study** |
|  |  | 19 | c.4956_4957insG | p.Glu1654X | NA | Ng et al. 2010 |
|  |  | 28 | c.6010C>T | p.Gln2004X | de novo | Ng et al. 2010 |
|  | **KB26** | **31** | **c.6295C>T** | **p.Arg2099X** | **NA,** de novo | **This study,** Ng et al. 2010 |
|  | **KB66** | **31** | **c.7246 C>T** | **p.Gln2416X** | **NA** | **This study** |
|  | **KB59** | **31** | **c.7903 C>T** | **p.Arg2635X** | **NA** | **This study** |
|  |  | 31 | c.7933C>T | p.Arg2645X | de novo | Paulussen et al. 2010 |
|  |  | 32 | c.8200C>T | p.Arg2734X | de novo | Paulussen et al. 2010 |
|  |  | 33 | c.8311C>T | p.Arg2771X | de novo | Paulussen et al. 2010 |
|  |  | 34 | c.8488C>T | p.Arg2830X | NA | Ng et al. 2010 |
|  |  | 34 | c.9961C>T | p.Arg3321X | NA | Ng et al. 2010 |
|  | **KB56** | **34** | **c.10135 C>T** | **p.Gln3379X** | **NA** | **This study** |
|  |  | 38 | c.10738C>T | p.Gln3580X | NA | Ng et al. 2010 |
|  | **KB46** | **39** | **c.10841C>G** | **p.Ser3614X** | **de novo** | **This study*** |
|  | **KB41, KB44** | **39** | **c.11119 C>T** | **p.Arg3707X** | **NA** | **This study** |
|  |  | 39 | c.11149C>T | p.Gln3717X | de novo | Ng et al. 2010 |
|  | **KB42** | **39** | **c.11269C>T** | **p.Gln3757X** | **NA** | **This study** |
|  | **KB25** | **39** | **c.11434C>T** | **p.Gln3812X** | **NA** | **This study** |
|  |  | 39 | c.11707C>T | p.Gln3903X | de novo | Paulussen et al. 2010 |
|  |  | 39 | c.11722C>T | p.Glu3908X | de novo | Paulussen et al. 2010 |
|  |  | 39 | c.11944C>T | p.Arg3982X | de novo | Paulussen et al. 2010 |
|  | **KB65** | **39** | **c.12076C>T** | **p.Gln4026X** | **NA** | **This study** |
|  |  | 39 | c.12241C>T | p.Gln4081X | NA | Ng et al. 2010 |
|  | **KB40** | **39** | **c.12274C>T** | **p.Gln4092X** | **NA** | **This study** |
|  |  | 39 | c.12697C>T | p.Gln4233X | de novo | Ng et al. 2010 |
|  |  | 39 | c.12703C>T | p.Gln4235X | NA | Ng et al. 2010 |
|  |  | 39 | c.13390C>T | p.Gln4464X | de novo | Ng et al. 2010 |
|  |  | 39 | c.13450C>T | p.Arg4484X | NA | Paulussen et al. 2010 |
|  |  | 40 | c.13580A>T | p.Lys4527X | inherited | Ng et al. 2010 |
|  |  | 40 | c.13606C>T | p.Arg4536X | NA | Ng et al. 2010 |
|  | **KB73** | **40** | **c.13666A>T** | **p.Lys4556X** | **NA** | **This study** |
|  |  | 48 | c.14710C>T | p.Arg4904X | NA | Ng et al. 2010 |
|  |  | 48 | c.14878C>T | p.Arg4960X | de novo | Paulussen et al. 2010 |
|  | **KB45, KB72** | **48** | **c.15079C>T** | **p.Arg5027X** | **NA,** de novo | **This study,** Paulussen et al. 2010 |
|  |  | 48 | c.15195G>A | p.Trp5065X | de novo | Ng et al. 2010 |
|  |  | 48 | c.15217C>T | p.Gln5073X | NA | Ng et al. 2010 |
|  |  | 48 | c.15618T>G | p.Tyr5206X | de novo | Ng et al. 2010 |
|  |  | 50 | c.16018C>T | p.Arg5340X | de novo | Paulussen et al. 2010 |
|  |  | 52 | c.16360C>T | p.Arg5454X | NA | Paulussen et al. 2010, Ng et al. 2010 |
|  |  | 53 | c.16501C>T | p.Arg5501X | NA | Ng et al. 2010 |
| **frameshift** | **KB75** | **4** | **c.472delT** | **p.Cys158ValfsX50** | **NA** | **This study** |
|  | **KB58** | 6 | **c.705delA** | **p.Pro235ProfsX26** | **NA** | **This study** |
|  | **KB57** | **8** | **c.1035_1036delCT** | **p.Leu345LeufsX18** | **NA** | **This study** |
|  |  | 10 | c.1301delT | p.Leu434GlnfsX496 | de novo | Paulussen et al. 2010 |
|  |  | 10 | c.1324delC | p.Pro442HisfsX487 | NA | Ng et al. 2010 |
|  | **KB89** | **10** | **c.1345_1346delCT** | **p.Leu449ValfsX5** | **NA** | **This study** |
|  |  | 10 | c.2110delG | p.Asp704ThrfsX226 | de novo | Paulussen et al. 2010 |
|  |  | 10 | c.2272delG | p.Glu758SerfsX171 | de novo | Paulussen et al. 2010 |
|  |  | 10 | c.2558_2559delCT | p.Arg853ProfsX3 | de novo | Paulussen et al. 2010 |
|  | **KB48** | **11** | **c.2993_2994insC** | **p.Pro998ProfsX70** | **de novo** | **This study** |
|  |  | 11 | c.3585_3586insA | p.Pro1196ThrfsX11 | de novo | Ng et al. 2010 |
|  |  | 11 | c.3889delC | p.Arg1297ValfsX33 | de novo | Paulussen et al. 2010 |
|  |  | 14 | c.4219_4222delTACT | p.Tyr1407ValfsX9 | de novo | Paulussen et al. 2010 |
|  |  | 25 | c.5585delA | p.Lys1862SerfsX14 | de novo | Paulussen et al. 2010 |
|  | **KB84** | **26** | **c.5779delC** | **p.Glu1927Lysfs120X** | **NA** | **This study** |
|  |  | 28 | c.5875_5891dup17 | p.Glu1965GlyfsX88 | NA | Ng et al. 2010 |
|  |  | 28 | c.5912delG | p.Ser1971ThrfsX76 | de novo | Paulussen et al. 2010 |
|  | **KB79, KB102** | **31** | **c.6595delT** | **p.Tyr2199IlefsX65** | **de novo**, de novo | **This study,** Ng et al. 2010 |
|  | **KB67** | **31** | **c.6638_6641delGCGC** | **p.Gly2213AlafsX50** | **de novo** | **This study** |
|  | **KB80** | **33** | **c.8273delG** | **p.Gly2758AlafsX29** | **NA** | **This study** |
|  |  | 34 | c.8641_8646delins | p.Arg2881AspfsX35 | NA | Paulussen et al. 2010 |
|  |  | 34 | c.9223dupT | p.Ser3075PhefsX3 | de novo | Paulussen et al. 2010 |
|  |  | 34 | c.9329delG | p.Arg3110ProfsX9 | NA | Paulussen et al. 2010 |
|  |  | 34 | c.9770dupA | p.Lys3250GlufsX43 | de novo | Paulussen et al. 2010 |
|  |  | 34 | c.10114_10126del13 | p.Ser3372CysfsX16 | NA | Paulussen et al. 2010 |
|  |  | 38 | c.10599_10630del32 | p.Val3534GlnfsX11 | NA | Ng et al. 2010 |
|  | **KB30** | **38** | **c.10606delC** | **p.Arg3536AlafsX122** | **NA** | **This study** |
|  | **KB101** | **39** | **c.11066_11078delCTGGATCCCTGGC** | **p.Ala3689ValfsX56** | **de novo** | **This study** |
|  |  | 39 | c.11497delC | p.Arg3833GlyfsX48 | de novo | Paulussen et al. 2010 |
|  |  | 39 | c.11794_11797delCAAC | p.Gln3932SerfsX46 | de novo | Ng et al. 2010 |
|  |  | 39 | c.12164_12165delCT | p.Pro4055ArgfsX6 | de novo | Paulussen et al. 2010 |
|  |  | 39 | c.12969dupA | p.Pro4324ThrfsX10 | de novo | Paulussen et al. 2010 |
|  | **KB54** | **39** | **c.13129_13130insT** | **p.Trp4377LeufsX33** | **NA** | **This study** |
|  |  | 48 | c.14845_14848dupCCTC | p.Leu4950ProfsX9 | de novo | Paulussen et al. 2010 |
|  |  | 48 | c.15444_15445delTT | p.Phe5149CysfsX9 | NA | Ng et al. 2010 |
|  | **KB64** | **53** | **c.16469_16470delAA** | **p.Lys5490ArgfsX21** | **NA** | **This study** |
| **indel** | **KB71** | **39** | **c.11819_11836dupTTCAACAACAGCAGCAGC** | **p.Lys3940_Gln3945dup**** | **inherited (M)** | **This study** |
|  | **KB77** | **48** | **c.15163_15168dupGACCTG** | **p.Asp5055_Leu5056dup** | **NA** | **This study** |
|  | **KB53** | **53** | **c.16489_16491delATC** | **p.Ile5497del** | **NA** | **This study** |
| **missense** | **KB32** | **11** | **c.3773G>A** | **p.Arg1258Gln** | **inherited (F)** | **This study** |
|  | **KB28** | **15** | **c.[4249A>G; 4252C>A]** | **p.[Met1417Val; Leu1418Met]** | **inherited (M)** | **This study** |
|  | **KB34** | **16** | **c.4565A>G** | **p.Gln1522Arg** | **inherited (F)** | **This study** |
|  | **KB27** | **34** | **c.8521C>A** | **p.Pro2841Thr** | **NA** | **This study** |
|  | **KB38** | **48** | **c.[15084C>G; 15100T>G]** | **p.[Asp5028Glu; Phe5034Val]** | **de novo** | **This study** |
|  | **KB76** | **48** | **c.15176A>C** | **p.His5059Pro** | **NA** | **This study** |
|  |  | 48 | c.15326G>T | p.Cys5109Phe | de novo | Ng et al. 2010 |
|  |  | 48 | c.15536G>A | p.Arg5179His (2x) | de novo | Ng et al. 2010 |
|  |  | 48 | c.15629A>G | p.Tyr5210Cys | de novo | Paulussen et al. 2010 |
|  |  | 48 | c.15641G>A | p.Arg5214His | NA | Ng et al. 2010 |
|  |  | 50 | c.16019G>T | p.Arg5340Lys | NA | Ng et al. 2010 |
|  | **KB17** | **50** | **c.16019G>A** | **p.Arg5340Gln** | **NA** | **This study** |
|  |  | 51 | c.16283G>A | p.Gly5428Asp | de novo | Paulussen et al. 2010 |
|  |  | 52 | c.16391C>T | p.Thr5464Met (3x) | NA | Ng et al. 2010 |
| **splice site** | **KB31** | **Intron 3-4** | **c.400+1G>A** |  | **NA** | **This study** |
|  | **KB20** | **Intron 3-4** | **c.401-3A>G** |  | **de novo** | **This study** |
|  |  | Intron 22-23 | c.5320-2A>G |  | NA | Paulussen et al. 2010 |
|  | **KB29** | **Intron 42-43** | **c.13999+5G>A** |  | **NA,** de novo | **This study,** Paulussen et al. 2010 |
|  |  | Intron 46-47 | c.14516-1G>C |  | NA | Paulussen et al. 2010 |
|  |  | Intron 47-48 | c.14644-2A>G |  | de novo | Paulussen et al. 2010 |

*KB44 mutation has been described elsewhere (Ejarque et al. in press)

** p. Lys3940_Gln3945dupLysGlnGlnGlnGlnGln

(2x) and (3x): mutation recurrent two and three times, respectively

**Table S3.** Repeats (underlined and highlighted in red) that might mediate micro-deletions, micro-insertion/deletions (indel), and micro-duplications in the *MLL2* gene

| **ID** | **Mutation** | **AA change** | **Sequence** | **Repeat** | **Rescue-ESE** | **Fas-ESS** |
| --- | --- | --- | --- | --- | --- | --- |
| KB75 | micro-deletion  c.472delT  Exon 4 | p.Cys158ValfsX50 | GCAGGAGGGCCCAGAACTATGTGGTGTGGACAAGGCCATC  GCAGGAGGGCCCAGAACTA:GTGGTGTGGACAAGGCCATC | direct repeat | no change | gain of one ESS |
| KB58 | micro-deletion  c.705delA  Exon 6 | p.Pro235fsX26 | GTGCAGTGTGTGAGGGGCCAGGGGAGTTGTGTGACCTGTT  GTGCAGTGTGTGAGGGGCC:GGGGAGTTGTGTGACCTGTT | direct repeat | no change | no change |
| KB57 | micro-deletion  c.1035_1036delCT  Exon 8 | p.Leu345LeufsX18 | GGTTTGAGAACTACTCTCTCTGTCACCGCTGTCACAAAGCC  GGTTTGAGAACTACTCTCT::GTCACCGCTGTCACAAAGCC | direct repeat | no change | no change |
| KB89 | micro-deletion  c.1345_1346delCT  Exon 10 | p.Leu449ValfsX5 | CCCACCTGAGGAGTCACCCCTGTCCCCACCACCTGAGGAA  CCCACCTGAGGAGTCACCC::GTCCCCACCACCTGAGGAA | direct repeat | no change | no change |
| KB48 | micro-insertion  c.2993_2994insC  Exon 11 | p.Pro998ProfsX70 | GACCCTGAGCCTGTCCCCCC_TATGATCCTTCCCCCATCTC  GACCCTGAGCCTGTCCCCCC**C**TATGATCCTTCCCCCATCTC | direct repeat | no change | no change |
| KB79, KB102 | micro-deletion  c.5779delC  Exon 26 | p.Glu1927LysfsX120 | ACTGCAGAGGCCCTTTCTTCAAGGTGGACTCCCTTTGGGCA  ACTGCAGAGGCCCTTTCTT:AAGGTGGACTCCCTTTGGGCA | direct repeat | gain of one ESE | no change |
| KB79, KB102 | micro-deletion  c.6595delT  Exon 31 | p.Tyr2199IlefsX65 | CCGCCCACCTATCCCCCCTATCCTAGTCCTACGGGGGCCC  CCGCCCACCTATCCCCCC:ATCCTAGTCCTACGGGGGCCC | direct repeat | no change | no change |
| KB67 | micro-deletion  c.6638_6641delGCGC  Exon 31 | p.Gly2213AlafsX50 | CGCAGCCCCCGATGCTGGGCGCCTCATCTCGTCCTGGGGCT  CGCAGCCCCCGATGCTGG::::CTCATCTCGTCCTGGGGCT | direct repeat | no change | no change |
| KB80 | micro-deletion  c.8273delG  Exon 33 | p.Gly2758AlafsX29 | CCCCCAAGCAAGCTGAGTGGCCCCATCCTGGGGCCAGGG  CCCCCAAGCAAGCTGAGTG:CCCCATCCTGGGGCCAGGG | direct repeat | no change | no change |
| KB30 | micro-deletion  c.10606delC  Exon 38 | p.Arg3536AlafsX122 | GAGCAGATTGGTGTACACCGCAAGTCCCGGAAGGCTCTGT  GAGCAGATTGGTGTACAC:GCAAGTCCCGGAAGGCTCTGT | direct repeat | loss of one ESE | no change |
| KB101 | micro-deletion  c.11066_11078delCTGGATCCCTGGC  Exon 39 | p.Ala3689ValfsX56 | CATTCTGGTGGGGCTGGATCCCTGGCTGGCCCTTCAGGGGG  CATTCTGGTGGGG::::::::::::TGGCCCTTCAGGGGG | direct repeat | loss of one ESE | gain of three ESSs |
| KB71 | micro-duplication  c.11819_11863dupTTCAACAACAGCAGCAGC  Exon 39 | p.Lys3940_Gln3945dup | TTCAACAGCAGCAACAGCAGCAGCAGCTTCAACAACAGCAGCAGCAACAG  TTCAACAGCAGCAACAGCAGCAGCAGCTTCAACAACAGCAGCAGC**TTCAACAACAGCAGCAGCAACAG** | direct repeat | gain of one ESE | no change |
| KB54 | micro-insertion  c.13129_13130insT  Exon 39 | p.Trp4377LeufsX33 | TAGCAAGGGTCTGGGACCTT_GGGATCCCCCAGACAACCTA  TAGCAAGGGTCTGGGACCTT**T**GGGATCCCCCAGACAACCTA | direct repeat | no change | no change |
| KB77 | micro-duplication  c.15163_15168dupGACCTG  Exon 48 | p.Asp5055_Leu5056dup | GCTGAACCTGGACCTGGACCTGTGGGTGCACCTCAACTGT  GCTGAACCTGGACCTGGACCTG**GACCTG**TGGGTGCACCTCAACTGT | direct repeat | no change | no change |
| KB64 | micro-deletion  c.16469-16470delAA  Exon 53 | p.Lys5490ArgfsX21 | CGAAGTCGTGACATTTGACAAAGAGGACAAAATCATCATC  CGAAGTCGTGACATTTGACA::GAGGACAAAATCATCATC | direct repeat | loss of four ESEs | no change |
| KB53 | micro-deletion  c.16489-16491delATC  Exon 53 | p.Ile5497del | AGAGGACAAAATCATCATCATCTCCAGCCGGCGAATCCCC  AGAGGACAAAATCATCATC:::TCCAGCCGGCGAATCCCC | direct repeat | loss of one ESE | no change |
