# Supplementary figures and images for: Mutation spectrum of MLL2 in a cohort of kabuki syndrome patients
Source: Orphanet J Rare Dis. 2011 Jun 9;6:38. doi: 10.1186/1750-1172-6-38 (PMC3141365; doi:10.1186/1750-1172-6-38)

## Slide 1
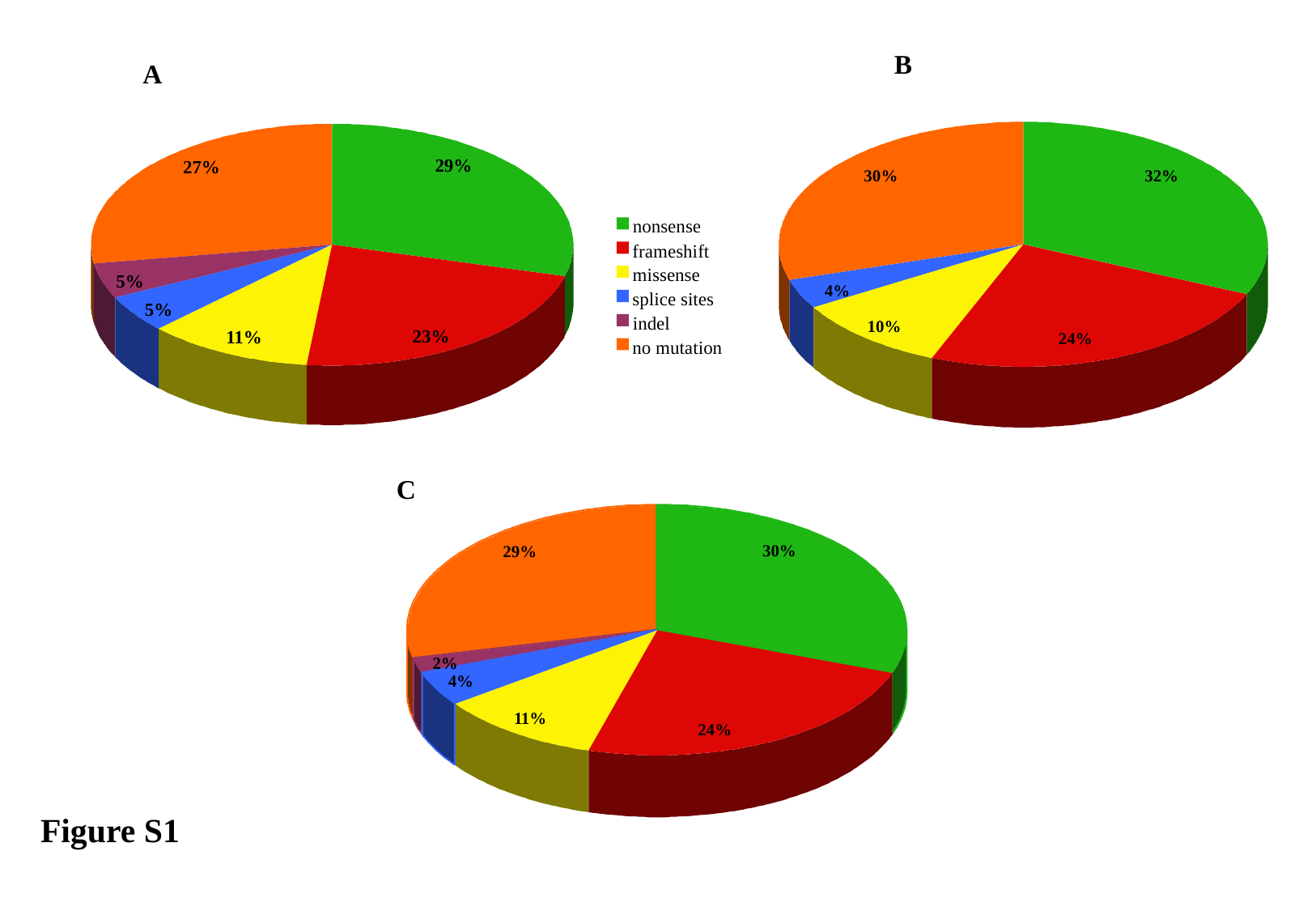

B
32%
30%
4%
10%
24%
A
29%
27%
nonsense
frameshift
missense
5%
splice sites
5%
indel
23%
11%
no mutation
C
30%
29%
2%
4%
11%
24%
Figure S1

Supplement: Additional file 2 — Figure S2. Frequency of different MLL2 mutation types in Kabuki syndrome patients identified to date. Our study (A), Ng et al. and Paulussen et al. studies (B), all three studies, A+B, (C). [file 1750-1172-6-38-S2.PPT]
